# Learning Hierarchical Representations with Spike-and-Slab Inception Network

**DOI:** 10.3390/s21196382

**Published:** 2021-09-24

**Authors:** Weizheng Qiao, Xiaojun Bi

**Affiliations:** 1 College of Information and Communication Engineering, Harbin Engineering University, Harbin 150001, China; qiaoweizheng@hrbeu.edu.cn; 2 College of Information Engineering, Minzu University of China, Beijing 100091, China

**Keywords:** convolutional neural networks, inception module, spike-and-slab units, dual variable operations

## Abstract

Recently, deep convolutional neural networks (CNN) with inception modules have attracted much attention due to their excellent performances on diverse domains. Nevertheless, the basic CNN can only capture a univariate feature, which is essentially linear. It leads to a weak ability in feature expression, further resulting in insufficient feature mining. In view of this issue, researchers incessantly deepened the network, bringing parameter redundancy and model over-fitting. Hence, whether we can employ this efficient deep neural network architecture to improve CNN and enhance the capacity of image recognition task still remains unknown. In this paper, we introduce spike-and-slab units to the modified inception module, enabling our model to capture dual latent variables and the average and covariance information. This operation further enhances the robustness of our model to variations of image intensity without increasing the model parameters. The results of several tasks demonstrated that dual variable operations can be well-integrated into inception modules, and excellent results have been achieved.

## 1. Introduction

The convolution neural network (CNN) significantly enhances the ability of image recognition by simulating the human brain. Traditional methods that mainly rely on artificial features result in a weak capacity to learn advanced feature information from the image. In contrast, CNN owns a powerful expression and generalization ability in analyzing and interpreting data. How to robustly learn effective representations from complex images of different sizes still remains unsolved. The research of image recognition based on CNN can significantly facilitate the development of artificial intelligence (AI) technology.

GoogLeNets [1], consisting of multiple inception modules, are typical and successful CNN in diverse applications [2,3,4]. It can generally be regarded as the initial tendency of the increasing width in the CNN. On the basis of the initial version, several well-known variants of the inception module soon appeared, such as inceptionv2/v3 [5] and inceptionv4 [6]. The above outcomes force insight into deep discriminative models. The question is whether we can improve the inception module to strengthen the capacity in the image recognition task, which has a wide prevalence in computer vision [7,8,9].

As a basic component for deep belief network (DBN) constructing, the restricted Boltzmann machine (RBM) has attracted much attention [10]. The capacity of the RBM will greatly affect the ability of deep probabilistic graphical models. How to improve their ability of representation learning has been studied extensively. The adoption of auxiliary variables can strengthen its ability to a certain extent, such as ssRBM [11,12,13]. In particular, the transition from RBM to ssRBM is like a transition from a conventional CNN to “Network in Network” in a deep discriminative model. Regarding further studying, how to utilize the mechanism of the transition from RBM to ssRBM for the modification of CNN models becomes a meaningful problem to be solved.

The ssRBM is characterized by the spike-and-slab units associated with each neuron in the hidden units. The slab variable is a real-valued vector obeying Gaussian distribution, while the spike variable is a binary variable obeying Binomial distribution. This strategy is efficient in capturing covariance information in image patches. This gives us inspiration to explore the application in CNN structures. In this paper, we introduce the spike-and-slab neurons to the modified inception module motivated by the operation in the ssRBM. In detail, we induced an element multiplication operation between the slab units and spike units in the convolutional layer, enabling our model to capture the average and covariance information simultaneously, further enhancing the capacity in learning hierarchical representations in the receptive field.

In this paper, we present two variants based on spike-and-slab variables with the inception module. First, we apply the spike-and-slab variables into a naive inception module referred to as origin ssIncep module. Secondly, we further explore the ssIncep module with dimensionality reduction, resulting in a novel block referred to as the proposed ssIncep module. It can also serve as the basic block for building deeper models. Finally, we built a different slab-and-spike inception network for diverse tasks by using the proposed ssIncep module, proving its superiority over the current-state-of-the-art models.

The remaining parts are organized as follows. Section 2 reviews the related work. Section 3 presents the details of our innovations. Section 4 is the experiments part. Further discussions are provided in Section 5. Section 4 is the conclusion.

## 2. Related Works

Deep discriminative models have witnessed impressive progress in the improvement of the model capacity and the applications in many domains in recent years. The major contributions are forced on the CNN variants from two perspectives. We highly discuss the related works associated with this aspect.

1. More powerful model capacity

One feasible approach to strengthening the capacity of the model is to increase the model complexity via deepening the models. Since LeNet5 [14] achieved excellent consequences in image recognition, researchers have proposed various prominent CNN models. The typical and influential networks are AlexNet [15], VGG [16], GoogleNet [1], ResNet [17], ShuffleNet [18], DenseNet [19] and SENet [20]. The network tends to be deeper for the purpose of learning abstract representations. The CNN models have developed to be particularly effective in image recognition. However, the basic module of existing CNNs is designed to capture single variable features, which are essentially linear features. This learning mechanism leads to a weak ability in feature expression, which further leads to insufficient feature mining. Researchers have solved this defect by deepening the network. The proposal of VGG proves that deepening the network is an effective means to improve the ability of feature expression. However, a deeper network easily leads to gradient dispersion, making the network not converge. When the number of layers is increased to 20 or more, the effect will be worse. In addition, the deep networks lead to parameter redundancy and overfitting, which leaves a great burden on training and reasoning. ResNet is evaluated to be an efficient network in alleviating gradient disappearances by tersely adding a shortcut edge during specific layers. However, the evaluation results showed that the improvement of precision achieved by deepening the network is getting smaller and smaller. All these efforts are aimed at increasing the structure of a model and obtaining a more powerful capacity.

2. Fewer computing requirements

Another method is to greatly reduce the model parameters and make the model tend to be lightweight. This greatly reduces the computing resources. The DenseNet similarly introduces shortcut mechanisms into the network structure, enabling information to flow during adjoining and specific disconnected layers. Furthermore, the DenseNet excellently reduces the scale of the model parameters by feature reusing and significantly alleviates the gradient vanishing. ShuffleNet utilizes the mechanism of sparse connection. By limiting the input of the convolution operation to each group, the computational burden of the model is significantly reduced. SENet uses global information to selectively enhance the beneficial feature channels and suppress useless feature channels, further improving the ability of feature learning.

Although the above-referred works focused on promoting the ability of feature learning and optimizing network configuration, how to learn robust representation and hierarchical representation from complex images is still unsolved.

In this paper, we selected the inception module derived from GoogLeNet as the basic structure for further modification. This scheme aimed at increasing the width of the network module, allowing each layer to learn abundant features, such as texture features with different directions and frequencies. The inception modules are closely related to grouping and multiscale strategies. In the naive version, we can regard the inception module as a special kind of multiscale learning. In the latter version of inception, the main contribution is the introduction of a 1 × 1 convolution operation. Furthermore, we introduce spike-and-slab variables into the inception module in order to capture the average and covariance information, promoting the performance of learning hierarchical representations excellently. This scheme can significantly promote the quality of learning representations while keeping the model parameters constant, thus maximizing the potential of the model capability. The modified inception module is an excellent candidate as a building block for developing more sophisticated models.

## 3. Materials and Methods

### 3.1. Motivations

**Figure 1:**
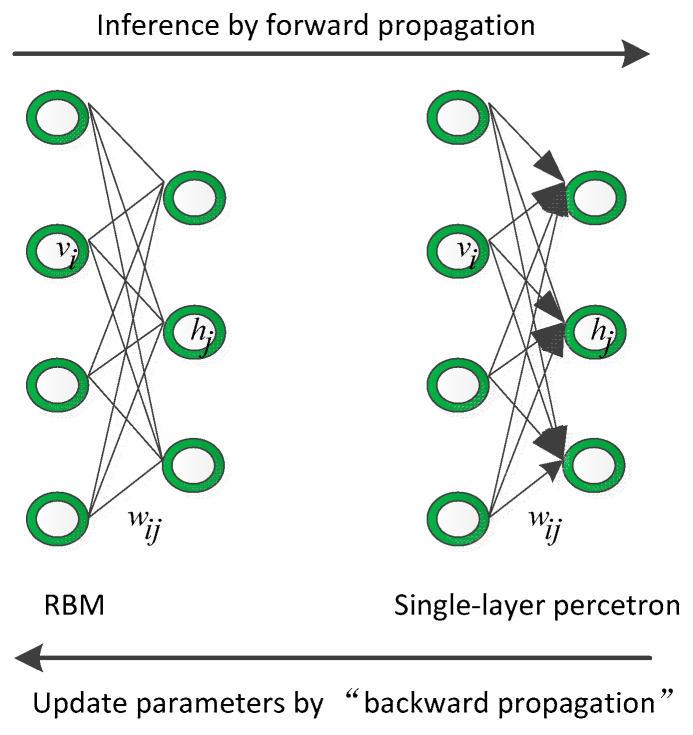
The relationship between the RBM and single-layer perceptron.

Reviewing the relationship between the innovation process of the probabilistic graphical model and the discriminative model is conducive to understanding the motivation of this paper. First, we discuss the relationship between the RBM and single-layer perceptron, as shown in Figure 1. The RBM is an undirected graph that owns a powerful modeling ability for any probability distribution. It is easy to infer given the input through forward propagation and updating the trainable parameters, they need negative samples through backward propagation. Considering the single-layer perceptron, it is a simple forward network that is easy to infer by forward propagation and updating the trainable parameters through backward propagation

**Figure 2:**
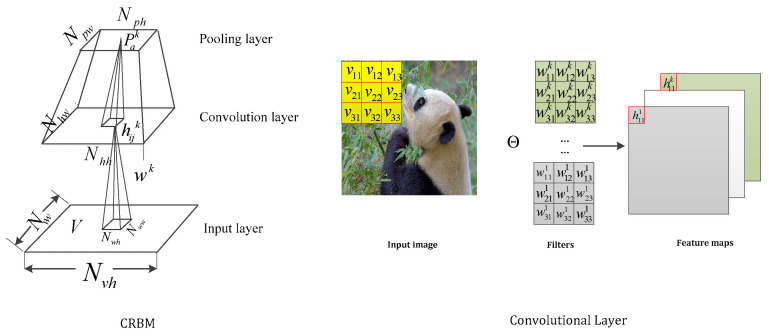
The relationship between the CRBM and convolutional layer.

When tracking with two-dimension images, the fully connected models such as RBM and single-layer perceptron are faced with huge parameter increments. This brings trouble in the modeling data. Then, a convolution operation is applied to the forward network. The basis of the CNNs is a convolutional layer that consists of diverse kernels and slides these kernels with a fixed stride to perform element-wise multiplication between the pixels and trainable kernel parameters. Motivated by this strategy, Lee [21] proposed the convolution restricted Boltzmann machine (CRBM) through inserting convolution operations into conventional RBM. As shown in Figure 2, this indicates the close relationship between the CRBM layer and convolution layer.

Generally, the traditional convolution layer has drawbacks and is incapable of modeling covariance patterns. Lin viewed the traditional convolutional layer as a generalized linear model (GLM) [22], resulting in the poor performance of nonlinearly separated data. In addition, the traditional convolutional layer cannot model covariance patterns reflecting the local stationary characteristics of each patch. In this case, ssRBM provides a pioneering thought by introducing slab-and-spike units to a deep neural network. It inspires us to modify the traditional convolution layer by combining a convolution operation with the spike-and-slab units. After reviewing the relationships between the probabilistic graphical models and the discriminative models, our motivation of this paper is clear to understand. We attempt to create a more powerful discrimination model by combining inception models with spike-and-slab units.

### 3.2. ssRBM Reviewed

**Figure 3:**
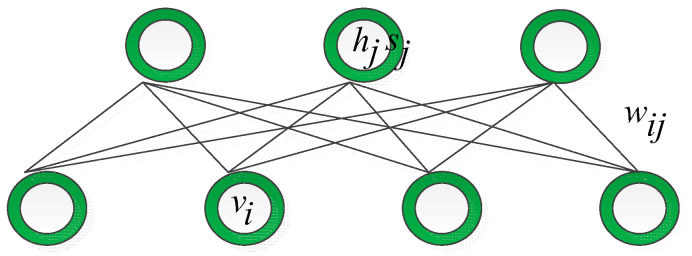
The structure of the ssRBM.

A typical RBM is a Markov random field with an undirected graph structure. In particular, the model consists of two layers with different characteristics. The visible layer owns binary variables representing the data, while the hidden layer consists of potential binary variables. For the purpose of modeling covariance structures existing in image patches, the ssRBM adopted Gaussian distribution in the units connected with binary spike variables and induced element-wise multiplication between two distinct variables [16]. The structure of the ssRBM is shown in Figure 3.

The study of the probability graph model can evolve into an exploration of the energy function. Therefore, we simply provided the energy function of ssRBM, as shown in Equation (1).

(1) $$E\left(v,s,h\right)=\frac{1}{2}v^{T}\Lambda v-\overset{N}{\underset{j=1}{\sum }}v^{T}W_{j}s_{j}h_{j}+\frac{1}{2}\overset{N}{\underset{j=1}{\sum }}s_{j}a_{j}s_{j}-\overset{N}{\underset{j=1}{\sum }}h_{j}b_{j}$$

where $v={\left[v_{1},v_{2},\ldots ,v_{N_{v}}\right]}^{T}\in {\mathbb{R}}^{N_{v}}$ is a vector with $N_{v}$ dimensions, Λ is a diagonal matrix for penalizing large values, N depicts the number of neurons in the hidden layer, $W_{j}$ refers to the j-th matrix weighting connection between the input layer and hidden layer with a size of $N^{v}\times N$, $s={\left[s_{1},s_{2},\ldots ,s_{N}\right]}^{T}\in {\mathbb{R}}^{N}$ and $h={\left[h_{1},h_{2},\ldots ,h_{N}\right]}^{T}\in {\left\{0,1\right\}}^{N}$ represent the slab variables and spike variables, respectively, and the $a_{j}$ is a real value penalizing a large value on $s_{j}$.

Through the reasoning of the energy function above, we can define the probability distribution of the ssRBM given v, h and s. The detailed formulations are shown in Equations (2)–(4).

(2) $$p(h|v)=\prod_{j=1}^{N}p(h_{i}=1|v)=\prod_{j=1}^{N}\sigma (\frac{1}{2}v^{T}W_{j}a_{j}^{-1}W_{j}{}^{T}v+b_{j})$$

(3) $$p(s|v,h)=\prod_{j=1}^{N}p(s_{j}|v,h)=\prod_{j=1}^{N}\mathbb{N}(\frac{1}{2}h_{j}a_{j}^{-1}W_{j}{}^{T}v+b_{j},a_{j}^{-1})$$

(4) $$p(v|s,h)=\prod_{i=1}^{N_{v}}p(v_{i}|s,h)=\prod_{i=1}^{N_{v}}\mathbb{N}(\Lambda^{-1}\sum_{i=1}^{N}W_{i}s_{i}h_{i},\Lambda_{i}^{-1})$$

where ℕ(a,b) represent the Gaussian distribution with a as the mean value and b as the variance value. Furthermore, we can infer the conditional distribution p(v|h) illustrated as follows.

(5) $$p(v|h)=\mathbb{N}(0,{\left(\Lambda -\sum_{i=1}^{N}W_{i}s_{i}a_{i}^{-1}W_{i}^{T}\right)}^{-1})$$

Equation (5) depicts the conditional matrix Gaussian distribution of *v*, while *h* is given. From this, we can infer that the ssRBM owns an ability to capture the covariance structure between observations.

Through the utilizing of dual latent variables, the ssRBM demonstrates a wondrous performance in data representation learning. In References [11,12,13], several vision tasks further proved the excellent robustness of the ssRBM to variations in the image intensity.

### 3.3. The Spike-and-Slab Inception Network Description

It retains great potential in promoting the robustness and the feature learning quality of the CNN model. In this paper, we proposed an improved inception module to explore the strategy of constructing an optimal local sparse architecture of a CNN by utilizing the available dense modules.

**Figure 4:**
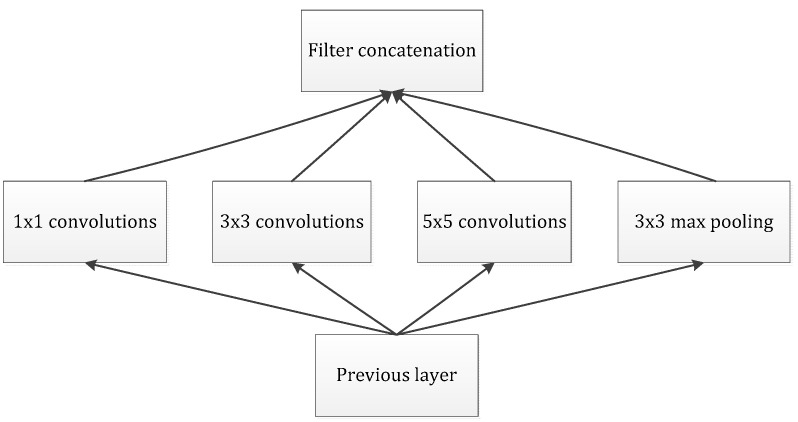
The naive version of the inception module.

Our goal is to build an improved neuron network, supplemented by the available convolution blocks. Our assignment was to devise an optimal local module and apply it to construct an integrated CNN structure, further preventing overfitting and reducing the use of computing resources. Arora [23] proposed a layer-by-layer architecture, which pays attention to the statistical correlation in the last layer and then performs clustering operations to obtain unit clusters with large correlations. Up to now, this structure has been regarded as a powerful module for building a CNN. This operation constructs the neurons of the next layer by connecting with the neurons of the previous layer. Assuming that the neurons in the previous layer correspond to the relevant areas of the original image, these neurons are regarded as filter banks. In the shallow layers, the related neurons tend to gather in partial areas. In this case, we can obtain a number of groups gathered in a unitary region, and then, we construct a 1 × 1 convolution layer and connect it to the current layer to cover them. Moreover, it can be inferred that, when the convolution is performed on a larger piece, a plurality of groups with larger spatial expansion characteristics would be covered, further leading to a decrease in the number of patches over larger and larger regions. To avoid patch alignment problems, the filter size of typical inception architecture is set to 1 × 1, 3 × 3 and 5 × 5, as shown in Figure 4.

The above design methodology is considered to be more convenient than necessary. It indicates that the advised structure is an association of all these layers, and the filter banks of each layer are finally concatenated into a single output vector, which further constitutes the input of the continuous module. In addition, the pooling mechanism has been proved to be a necessary operation for maintaining the effective performance of CNNs. It should be efficacious to design an alternative parallel channel with a pooling operation and apply it to each module. In view of the fact that “inception modules” are stacked and connected in a series, the output-related statistics of each module will definitely change, which leads to a decrease of the spatial concentration, which is mainly due to representations of higher abstractions learned by higher layers. This suggests an increase in the ratio of 3 × 3 and 5 × 5 convolutions while focusing on deeper layers. However, the naïve version of the inception module would face the heavy burden of dealing with too many parameters due to the multichannel filters.

**Figure 5:**
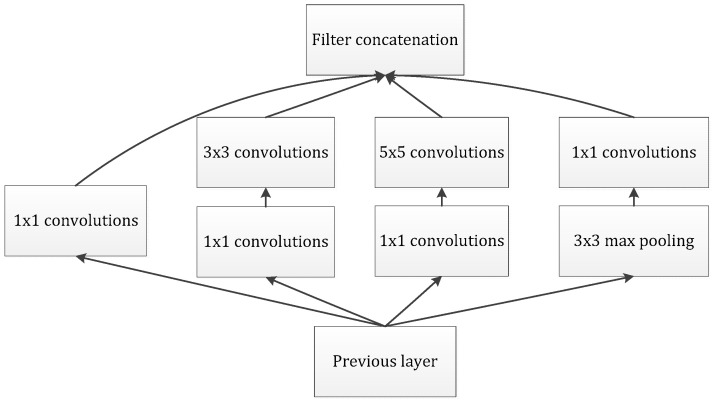
Inception module with dimensionality reduction.

In Figure 5, it describes the inception module with dimension reduction, which employs 1 × 1 convolution to reduce the dimensionality wisely for the purpose of reducing the demand of the computing resource. In our method, we adopted the inception module with dimensionality reduction to assist our network.

Furthermore, we also introduced transition mechanisms from the RBM to ssRBM to improve our inception architecture. In order to characterize a convolution operation, we considered binary variable spike units and real-valued slab units in the hidden layers. We applied binary variables and real-valued units to the convolution kernel instead of the original activation functions, such as ReLU and Sigmoid. Generally, the conventional convolution operation is described in Equation (6):

(6) $$Conv\_out=g(y)=g\left(\sum_{i}\left(\alpha *w_{i}\right)+b_{i}\right)$$

where α denotes the input of the current convolution layer, $w_{i}$ denotes the weight matrix of kernel i, $b_{k}$ represents the bias variable, ∗ denotes the convolution operation and g represents the nonlinear activation function, which could be ReLU or Sigmoid.

Here, we provide the operation process of a spike-and-slab operation based on conventional convolution operation. Equation (7) depicts the acquirement of the binary variable spike value represented by h_feature, where *sigm* represents a logical sigmoid.

(7) $$h\_feature=sigm\left(\frac{1}{2}\sum_{i}(\alpha *w_{i})\cdot \sum_{i}(\alpha *w_{i})+b_{i}\right)$$

Thus, we can obtain a real-valued slab represented by s_feature, illustrated in Equation (8):

(8) $$s\_feature=\sum_{i}(\alpha *w_{i})\cdot h\_feature$$

Finally, the output of spike-and-slab convolution layer can be obtained through an element multiplication operation between the slab units and spike units.

**Figure 6:**
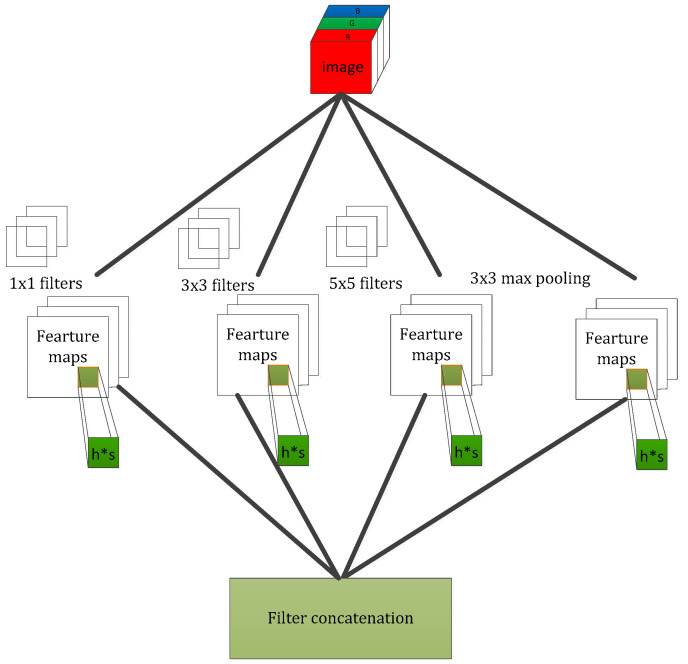
The graphic representation of the local structure in the spike-and-slab Inception module.

Figure 6 shows the graphic representation of the local structure enhanced by two variables (h∗s) without dimension reduction. This operation greatly enhances the capability of feature learning in image patches, enabling our model to capture the average and covariance information simultaneously. In this paper, our proposed spike-and-slab inception network contains several modules of the type depicted in Figure 6 that are assisted by a 1∗1 convolution operation for dimensionality reduction, and the modules are stacked upon each other in sequence. We stacked the spike-and-slab modules in the deeper layers, and the shallower layers consisted of a spike-and-slab convolution layer and pooling operation. It is worth noting that we designed the spike-and-slab inception network with different structures for various version classification tasks, and the details of the specific network configuration will be depicted in Section 4.

Moreover, the training of our proposed model might encounter an internal covariate shift due to the output data characteristic of spike-and-slab units being hard to optimize. Therefore, we introduced batch normalization into our module, which enabled us to adopt higher learning rates and pay less attention to network initialization in the training process.

## 4. Results

In this part, we mainly evaluate the advanced nature of our proposed spike-and-slab inception network (ssIncep-Net) by conducting a series of experiments qualitatively and quantitatively. Various kinds of representative datasets, including the fashion-mnist dataset, ILSVRC2012 classification dataset, CIFAR-10 dataset, CIFAR-100 dataset, Caltech-101 dataset and Caltech-256 dataset, were selected for model performance detection. The experiments were specifically divided into two broad categories, of which the first part was to validate the model performance through classification tasks on the dataset above, while the other part explored the quality and robustness of the learned features in the variations of input through the feature visualization exhibition.

Finally, our experiments were performed in the Ubuntu 16.04 system. In addition, we adopted two GTX2080 GPUs to accelerate computing platforms, and our codes were written by Pylearn3 based on Tensorflow [24].

### 4.1. Classification Tasks for Quantitatively Analysis

#### 4.1.1. Caltech-101 and Caltech-256 Classification

Caltech-101 and Caltech-256 were adopted in this experiment to quantitatively investigate the superiority of our proposed method on the learning of hierarchical representations. Caltech-101 is an authoritative public dataset that is mainly selected from the Google Image Dataset. It is composed of 9146 object pictures belonging to 102 categories (one background). Caltech-256 was evolved and enhanced by Caltech-101. Inconsistent categories were manually removed. There are 30,607 images belonging to 257 image categories (one background) existing in the dataset, and each category contains more than 80 images. During our research, images of both datasets were resized to 200*200 resolution before the training. It should be pointed out that, for Caltech-101, we randomly select 30 images for training in each class and up to 50 images for verification in each class, which is consistent with the authoritative approach. In addition, we randomly select 60 images for training and verification in each class at Caltech-256.

In this part, we propose our specific ssIncep-Net by improving and optimizing the structure of Inception-V1 (GoogLeNet). The configuration of ssIncep-Net used for Caltech-101 and Caltech-256 classification is shown in Table 1. From Table 1, we can discover that the network is mainly composed of two convolution layers and seven spike-and-slab inception blocks named ssinception 3a, ssinception 3b, ssinception 4a, ssinception 4b, ssinception 4c, ssinception 5a and ssinception 5b, with the convolution layers assisted by a dual variable mechanism and batch normalization.

The classification tasks in Caltch-101 and Caltech-256 were performed by ssIncep-Net with the same specific structure and different hyperparameter. The details of the training parameters are illustrated as follows. The batch sizes of the Caltch-101 and Caltech-256 recognition tasks were set to 24 and 32, respectively. We utilized momentum as the training model optimizer with epochs of 300 for the whole network. The weight decay of Caltch-101 and Caltech-256 recognition tasks was 0.0001 and 0.0003, respectively. The learning rate of both tasks was set to 0.001.

We evaluated the property of our proposed model compared with several baseline methods. Specifically, Incep-Net and our ssIncep-Net share consistent structures, except that Incep-Net employs a conventional convolution operation. Recognition accuracy of the Caltech-101 and Caltech-256 is depicted in Table 2 and Table 3, from which we can observe that ssIncep-Net is superior to Incep-Net in the classification accuracy of both datasets, indicating that ssIncep-Net owns a more effective ability in extracting abstract features compared with Incep-Net. This can be explained by the particularity of our model in capturing covariance features and learning hierarchical representations from the image patch. Furthermore, several state-of-the-art methods were selected in experiments for the purpose of verifying the advancement of our method. The results showed that ssIncep-Net performs better than general convolution networks.

**Table 2:** Recognition accuracy on the Caltech-101.

Method	P. Li [25]	Bo L [26]	Li [27]	Chao [28]	Zeiler [29]	IncepNet	ssIncep-Net
**Accuracy**	78.46%	81.4%	83.0%	84.3%	86.5%	85.31%	88.67%

**Table 3:** Recognition accuracy on the Caltech-256.

Method	Li [27]	Lei [30]	Zhou [31]	Zheng [32]	Zeiler [29]	Incep-Net	ssIncep-Net
**Accuracy**	51.9%	58.17%	65.1%	64.06%	72.7%	69.58%	74.82%

#### 4.1.2. Classification on ILSVRC2012

In this part, we verify our proposed model by performing the classification tasks on the ILSVRC2012 classification challenge dataset. The dataset involves the task of classifying images from a dataset of 1000 classes in which 1.2 million images were used for training and another 150 thousand images were used for validation and testing.

In our experiment, our ssIncep-Net is based on the standard Inception-V4. In view of the huge scale owned by ILSVRC2012 and the tremendous requirement of computing the resources, an efficient method has been adopted for facilitating the experiment and convenient verification on our model. In general, ssIncep-Net can be regarded as a specific structure that shares the same configuration network with Incep-Net, except that ssIncep-Net is assisted by the dual variables mechanism. Therefore, we utilized the pretraining model of Inception-V4 on ILSVRC2012, which is an open source on Github, and upgrade Inception-V4 with a dual variable mechanism to build our specific ssIncep-Net. The updated model shares the same parameters and structure with the original Inception-V4. We performed training and validating on the two models. Furthermore, a fine-tuning operation was also carried out in order to optimize the parameters of ssIncep-Net, making it more suitable for a spike-and-slab operation in the convolution kernel. We randomly selected 500 images per class from ILSVRC2012 for fine-tuning; all of them were resized to 224*224.

The Top-5 and Top-1 accuracies were adopted as the evaluation indicators, and the results are shown in Table 4. It demonstrates that ssIncep-Net owns better performances than the original Inception-V4 and achieved 1.05% and 0.37% improvement of Top-1 and Top-5, respectively. It reveals the effective ability of ssIncep-Net in learning covariance information and hierarchical representations in the image patches. Furthermore, several competitive methods were selected in the experiment for the purpose of verifying the advancement of our method. It is worth noting that we introduced SENet, which is one of the advanced CNN models, achieving ILSVRC2017. We adopted SENet-154 for further verification on the modern architecture. In detail, we introduced spike-and-slab units to SENet-154 and verified the validity of the dual-variable mechanism in the new model. Similar with the pretraining process conducted in ssIncep-Net, we acquired the pretraining model of SENet-154 on Github and took it as the initialization parameters for SENet-154 with spike-and-slab units. Then, we fine-tuned the model using the methods described above.

From Table 4, we can infer that SENet-154 with spike-and-slab is slightly superior to the original SENet-154 and achieved 0.56% and 0.19% improvement of Top-1 and Top-5, respectively. This further verified the superiority of SENet-154 with spike-and-slab, demonstrating that the spike-and-slab mechanism and considering more about high-dimensional robustness features. It is worth noting that the performance of SENet-154 is better than that of ssIncep-Net due to the inherent limitations of inception modules, which are not effective compared with the squeeze-and-excitation blocks in SENet. In conclusion, despite the fact that improvements of the error rates were subtle, it can still prove the validity of the innovation in CNNs. Furthermore, our intention was to explore a new perspective to enhance the ability of CNNs and provide researchers with a new idea to improve the latest models.

**Table 4:** Recognition error rates on ILSVRC2012.

Method	Error Rate (%)	
	Top-1	Top-5
Krizhevsky [15]	40.7	18.2
Sermanet [33]	35.74	14.18
Howard [34]	37.0	15.8
Zeiler [29]	37.5	16.0
Inception-V4 [6]	20.0	5.0
ssIncep-Net	18.95	4.63
SENet-154 [20]	18.68	4.47
SENet-154 with spike-and-slab	18.12	4.28

#### 4.1.3. Classification on Fashion MNIST Variants

This part evaluates our proposed method for classification tasks in Fashion MNIST and its variation datasets, further confirming the preponderance of ssIncep-Net quantitatively. The Fashion MNIST dataset is a dataset containing 10 categories of fashion products, which is composed of 60,000 training images and 10,000 test images. The resolution of each image is 28 × 28. The raising of this dataset is to replace the original MNIST dataset, further benchmarking the machine learning and relevant algorithms.

**Figure 7:**
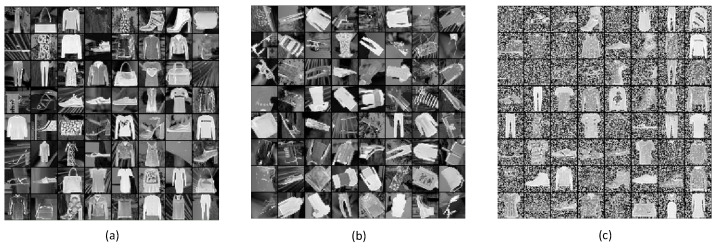
Visualizations of samples from different variants of Fashion MNIST. (**a**) Fashion-mnist-back, (**b**) Fashion-mnist-rot-back and (**c**) Fashion-mnist-back-rand.

Further, we created three variants by corrupting the Fashion MNIST dataset in the corresponding strategy of model verification. Our idea on variations generating was deeply affected by Reference [35]. In detail, the dataset variations contain Fashion-mnist-back, Fashion-mnist-rot-back and Fashion-mnist-back-rand, all of which are depicted in Figure 7a–c and described as follows.

Fashion-mnist-back: utilizing random black and white images as the background for original Fashion MNIST data.

Fashion-mnist-rot-back: adding rotating operations with a random angle for Fashion-mnist-back data.

Fashion-mnist-back-rand: adding random Gaussian noise as the background for original Fashion MNIST data.

For each variant, 60,000 images were obtained, of which 46,000 samples were designated for training. Thus, we can conduct comprehensive experiments belonging to different difficulty levels, arguing the excellent performance of our method persuasively.

In this task, the configuration of our particular ssIncep-Net is shown in Table 5. As a baseline method, the Incep-Net shares the same structure with ssIncep-Net. Both Incep-Net and ssIncep-Net are assisted with batch normalization. In addition, we built several models as related baseline methods and performed them in this task. The detailed framework and network parameters of the baseline methods are described as follows:

DBN-3: We stacked four RBMs to construct a DBN model in which a Gaussian-Binary RBM is utilized as the first layer for the purpose of capturing a continuous value in the images while two binary RBMs are selected to construct the remaining two layers, with 512, 512 and 128 neurons existing in each layer to perform an excellent performance. In addition, we concatenate a SoftMax layer to the last hidden layer to make a final prediction. We applied a pretraining strategy to the DBN model, which can enable the model to optimize the initial network parameter toward the optimum values. In order to overcome the overfitting, we adopted a batch stochastic gradient descent to fine-tune the DBN-3 with the assistance of L2 regularization. The specific architecture of DBN-3 keeps consistent with the default in Reference [35].

GDBM-2: GDBM is proposed by Reference [36] based on DBM. In this paper, we utilized a GDBM-2 model with the same hyperparameter setting as the model in Reference [36].

NIN: The structure of NIN adopted in this paper kept consistent with that adopted in Reference [21] on the cifar-10 classification task. In addition, we utilized the specific setting of the model parameters, which are open access and exist in the caffe model zoo.

ResNet-10: ResNet is a well-performed model in the domain of image classification [17]. The application structure in this paper owned three basic blocks. Each block consisted of 3 × 3 “same” convolutions, 3 × 3 “same” convolutions and 1 × 1 convolutions followed by 2 × 2 max pooling.

Table 6 lists the recognition error rates of our methods compared with Incep-Net and other state-of-the-art methods. We can reveal that ssIncep-Net surpassed the comparable methods and achieved the best performance on the classification task of the Fashion-MNIST variations. This indicates that our proposed method is more robust with the different variations between the datasets. Summarizing the reasons, it is mainly due to the mechanisms of the spike-and-slab variables adopted in our model. The innovation efficiently improves the performance of ssIncep-Net in learning hierarchical representations existing in the variations.

#### 4.1.4. Classification Task on CIFAR-10 and CIFAR-100

In this part, we quantitatively evaluate the performance of ssIncep-Net using the CIFAR-10 and CIFAR-100 datasets. The reason why we chose these datasets is that they have proven to be more complex and involve various features compared with Fashion MNIST, thus verifying our method more scientifically. The structures of the ssIncep-Net and baseline Incep-Net applied in this section are the same as that in the classification task of the Fashion MNIST variants. We also specified DBN-3 and ResNet-10 as the additional baseline methods, and their detailed structures are shown above.

Table 7 describes the recognition error rates on CIFAR-10 and CIFAR-100, which indicates that ssIncep-Net outperforms other methods on these two datasets. We can conclude that ssIncep-Net is more effective in modeling abstract features and covariance information.

**Table 7:** Recognition error rates on CIFAR-10 and CIFAR-100.

Method	Dataset	
	Cifar-10	Cifar-100
DBN-3	14.32	47.53
ResNet-10	9.36	42.46
HighwayNet [37]	10.84	32.40
Guoan Yang [38]	8.44	35.41
Benteng Ma [39]	10.68	33.23
Incep-Net	8.12	30.84
ssIncep-Net	5.91	28.60

### 4.2. Visualization Verification for Qualitatively Analysis

#### 4.2.1. Learning Hierarchical Representations from Caltech-256

For further investigation, a visualized operation was adopted to qualitatively explore how our model operated. In this part, we conducted a visualized operation on the well-trained ssIncep-Net and Incep-Net obtained from the previous classification task on Caltech-256. The object category of the airplanes was utilized for the visualization of hierarchical features. Before visualization, the selected image was resized to 200∗200. Our visualization operation was carried out in two diverse aspects.

**Figure 8:**
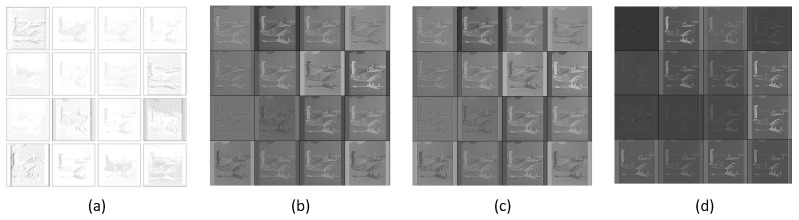
Visualization of feature maps in the first convolutional layer. (**a**) Feature maps by Incep-Net, (**b**) hybrid feature maps by ssIncep-Net, (**c**) slab feature maps by ssIncep-Net and (**d**) spike feature maps by ssIncep-Net.

First, we visualized the feature maps generated by a specific layer. Figure 8 shows the visualization results. The feature maps generated from the first convolution layer of Incep-Net are illustrated in Figure 8a, while Figure 8b–d shows the learned hybrid feature maps (element-wise multiplication between the slab features and spike feature maps), slab feature maps and spike feature maps obtained from the first modified convolutional layer of ssIncep-Net, respectively.

Figure 8b–d shows that our model can extract more details of airplanes than Incep-Net in Figure 8a. The feature maps of the airplanes shown in Figure 8b–d present more detailed features of the image edge significantly and reveal more distinct objects. However, the feature maps in Figure 8a are obviously blurry and lack details, and some objects in Figure 8a are even mixed up with the background and cannot be distinguished. This demonstrates the remarkable ability owned by our model in learning more detailed representations of image edges and object parts. In addition, it is worth noting that the learned spike features can blur partial structures of images to a certain extent, further resulting in fuzzy areas in feature maps, as shown in Figure 8c, despite the fact that the spike features of ssIncep-Net facilitate the integration of effective information and contribute to extracting slab features.

**Figure 9:**
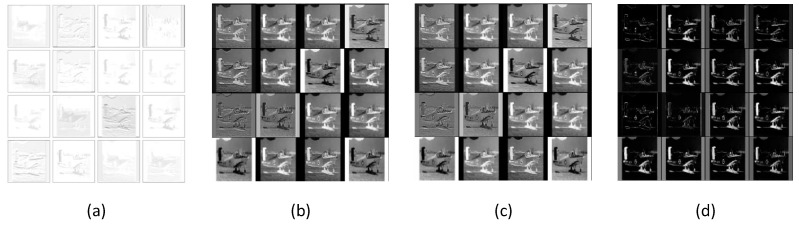
Visualization of feature maps in the first pooling layer. (**a**) Feature maps by Incep-Net, (**b**) hybrid feature maps by ssIncep-Net, (**c**) slab feature maps by ssIncep-Net and (**d**) spike feature maps by ssIncep-Net.

Our model utilized dual variable max-pooling to replace the classic pooling operations and implemented the new mechanism in the first pooling layer of our model, through which we obtained the corresponding pooling feature maps depicted in Figure 9. Figure 9b–d exhibits different types of pooling feature maps, containing a hybrid feature, slab feature and spike feature, respectively. Compared with the pooling feature maps obtained from the first pooling layer of the Incep-Net depicted in Figure 9a, the feature maps in Figure 9b–d present more detailed features of objects that are clearer. This is mainly due to the fact that the specific dual variables strategy can extract more abstract features. It further demonstrates that our model can learn more effective representations and pay more attention to translation-invariant features.

**Figure 10:**
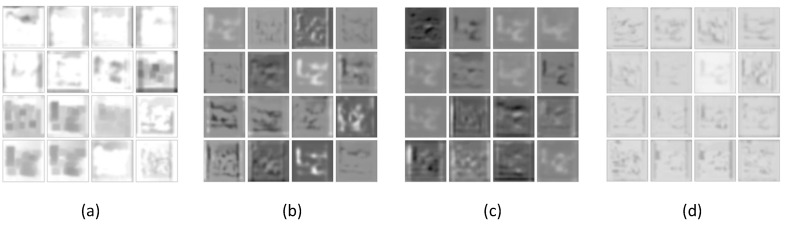
Visualization of feature maps in the Inception 3a and ssInception 3a layers. (**a**) Feature maps by Incep-Net, (**b**) hybrid feature maps by ssIncep-Net, (**c**) slab feature maps by ssIncep-Net and (**d**) spike feature maps by ssIncep-Net.

Furthermore, we visualize the feature maps generated by ssInception 3a and Inception 3a shown in Figure 10. These operations mainly verify the availability of the dual variable mechanism combined with the inception module. From Figure 10, we can conclude that the features extracted from the deeper layer become sparser. As shown in Figure 10b–d, the edge information of airplanes is well-preserved by ssIncep-Net, while the information in Figure 10a is extremely fuzzy or even gone. This strongly proves the effectiveness of the dual variable mechanism combined with the inception module. It further reveals that our proposed method is more efficient in learning abstract features and extracting hierarchical invariant representations compared with Incep-Net.

**Figure 11:**
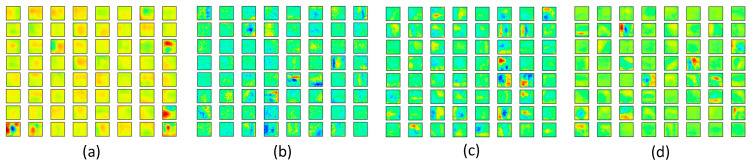
Visualization of convolution kernels in the first convolutional layer. (**a**) General convolution kernels by Incep-Net, (**b**) hybrid convolution kernels by ssIncep-Net, (**c**) slab convolution kernels by ssIncep-Net and (**d**) spike convolution kernels by ssIncep-Net.

Secondly, we visualized the learned convolution kernels in the first convolution layer of ssIncep-Net and IncepNet; both owned 15*15 convolution kernels in 64 channels. In order to visualize the convolution kernel more intuitively, filters with much larger sizes were utilized. The results are illustrated in Figure 11.

Figure 11a–d depicts the general convolution kernels, hybrid convolution kernels, slab convolution kernels and spike convolution kernels, respectively. It can be inferred from Figure 11 that the training convolution kernels in the visualization layer mainly reflect the selective lineaments, feature angles and the surface margin of the image. The visualized convolution kernels shown in Figure 8b present more detailed features of the edge and angles information. However, almost all of the visualized convolution kernels in Figure 11a are blurry and lack detailed information. This demonstrates that our model can learn more edge and angles information compared with the general convolution kernels in Figure 11a, which is mainly due to the ability of the slab hidden units in extracting covariance information in the image patches. This further proves that a dual variable mechanism efficiently promotes the performance of Incep-Net.

#### 4.2.2. Visualization on Embedding Representations Learned from Cifar-10

Moreover, we conducted a series of visualization operations on embedding representations, qualitatively verifying the performances of the specific layers existing in our model. We selected Cifar-10 for visualization due to two factors: Cifar-10 is more complex than Fashion MNIST, involving various features. It contains 10 categories, which is a suitable proportion for visualizing the distribution of each category compared with Cifar-100. Similar to the previous section, we visualized hierarchical representations of the well-trained Incep-Net and ssIncep-Net obtained from the previous classification task in Cifar-10.

By utilizing dimensionality reduction algorithms, the high-dimensional representation learned from specific layers of Incep-Net and ssIncep-Net is embedded into the two-dimensional space, thus qualitatively verifying the robustness of our model. In this paper, we adopted t-SNE to perform dimensionality reduction, which was proven to be an efficient visualization tool compared with PCA. High-dimensional data was gathered from the output of specific layers. We revealed inherent attributes of the network through a layer-by-layer analysis.

**Figure 12:**
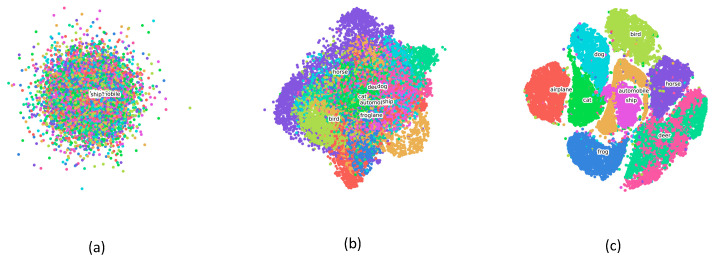
Image representation visualization of Incep-Net performed by t-SNE. (**a**–**c**) The output of the second convolutional layer, Inception 3b block and SoftMax, respectively.

**Figure 13:**
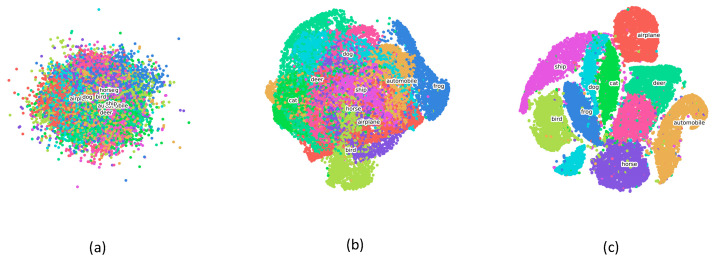
Image representation visualization of ssIncep-Net performed by t-SNE. (**a**–**c**) The output of the second convolutional layer, ssInception 3b block and SoftMax, respectively.

The embedding results of Incep-Net and ssIncep-Net are depicted in Figure 12a–c and Figure 13a–c, respectively. In detail, Figure 12a–c represented the output of the second convolutional layer, Inception 3b block and SoftMax existed in Incep-Net, while Figure 13a–c represented the output of the second convolutional layer, ssInception 3b block and SoftMax existed in ssIncep-Net, respectively.

Through the observations between Figure 12a,b and Figure 13a,b, we can infer that our ssIncep-Net achieved a better performance in the corresponding layer compared with Incep-Net, mainly due to the spike-and-slab operation adopted in our proposed model. The comparison between Figure 12c and Figure 13c revealed that ssIncep-Net is efficient in classification and recognition by stacking a specific ssIncep module. The representations acquired by deeper layers turned out to be more abstract and discriminated compared with the shallower layers, further verifying the efficiency of the proposed ssIncep-Net in dealing with the version classification task.

## 5. Discussion

In this paper, we explored an improved CNN structure with an inception module, which can perform better in feature mining without increasing the computational burden. We conducted the experiment in two aspects, classification tasks for a quantitative analysis and visualization verification for a qualitative analysis. Based on the results of a series of classification tasks on various datasets, the quantitative analysis tasks efficiently explained the progress of our proposed ssIncep-Net from the view of recognition accuracy. Furthermore, the qualitative analysis was devoted to exploring the interpretability of our improvement on the inception module using visualized manipulation. Compared with References [36,37,38], the integration of the two aspects in this paper comprehensively proved the effectiveness and interpretability of our model. In addition, the reviewing of the relationships between the innovation process of the probabilistic graphical models and discriminative models led to better interpretability in theory for our innovation. Through the ingenious operation of spike-and-slab units, we finally demonstrated the extraordinary performance of ssIncep-Net in learning hierarchical representations from images.

Furthermore, we conducted a complexity analysis to better discuss ssIncep-Net. Considering the whole model, the complexity of ssIncep-Net is $ο(\sum_{l=1}^{D}M_{l}^{2}\cdot K_{l}^{2}\cdot C_{l-1}\cdot C_{l})$, in which l denotes the lth layer of the model, D denotes the number of convolution kernels that the model owns, M and K indicate the side length of the feature maps and convolution kernels, respectively. $C_{l-1}$ and $C_{l}$ denote the number of input channels and output channels in each layer, respectively. It revealed that the complexity of our model was the same as that of Incep-Net. Our model showed better performance under the premise of the same complexity, further indicating the advancement of our model. Compared with the conventional network, ssIncep-Net can achieve the same performance with fewer network layers, proving the advantages of the network in computing and memory. However, our approach encountered difficulties in training, specifically reflected in harder convergence and larger training epochs. This was mainly due to the output information characteristic of spike-and-slab units being difficult to optimize. Batch normalization partially solved this problem, but it was not perfect. Considering that the optimize inception structure is an inherent problem, we need to observe the problems existing in the gradient transmission of the model. We suggest researching further improvements of the regularization schemes to optimize the training mechanism and energy function, making the distribution of the energy function flatter in higher dimensional spaces. This also provides a research direction for us and other researchers to study. On all accounts, the spike-and-slab inception module proposed by us is a remarkable candidate and can be utilized as a building block for researching more sophisticated models. We look forward to building more advanced CNN architecture based on this work, further exploring a better strategy for solving the confrontation problem.

## 6. Conclusions

In this paper, we introduced a novel deep discriminative model by the improvement of deep convolutional neural networks (CNN) with an inception module for image recognition tasks. Our proposed ssIncep-Net consists of two parts, including an improved discriminative deep convolution inception module and dual variable operations, which further enable it to be well-performed. Our model was more efficient compared with the conventional model due to the spike-and-slab features embedded in the convolution, which can capture higher-order and hierarchical features. In addition, our work is different from other works, since we seriously control the size of the model and number of parameters to overcome overfitting. This operation further enhances the robustness of our model for variations in image intensity.

The experiment results compared with several other methods indicated that spike-and-slab units can be well-integrated into inception modules and reach excellent achievements. Furthermore, this work will help to explain the superiority of the inception module from the view of improving the activation function and guiding the researchers to design new structures.

## Figures and Tables

**Table 1:** The configuration of ssIncep-Net used for Caltech-101 and Caltech-256 classification.

Layers	Configuration for Each Layer
The First Convolutional Layer	size of kernels (64,15,15) with a stride of 2
Pooling Layer	size of pooling (3,3) with a stride of 2
The Second Convolutional Layer	size of kernels (64,3,3) with a stride of 1
Pooling Layer	size of pooling (3,3) with a stride of 2
The ssInception 3a block	size of kernels (64,1,1), (128,3,3) and (32,5,5) and size of pooling (3,3)
The ssInception 3b block	size of kernels (128,1,1), (192,3,3) and (96,5,5) and size of pooling (3,3)
Pooling Layer	size of pooling (3,3) with a stride of 2
The ssInception 4a block	size of kernels (192,1,1), (208,3,3) and (48,5,5) and size of pooling(3,3)
The ssInception 4b block	size of kernels (128,1,1), (192,3,3) and (96,5,5) and size of pooling(3,3)
The ssInception 4c block	size of kernels (246,1,1), (320,3,3) and (96,5,5) and size of pooling(3,3)
Pooling Layer	size of pooling (3,3) with a stride of 2
The ssInception 5a block	size of kernels (128,1,1), (192,3,3) and (96,5,5) and size of pooling(3,3)
The ssInception 5b block	size of kernels (192,1,1), (256,3,3) and (128,5,5) and size of pooling(3,3)
Pooling Layer	size of pooling (3,3) with a stride of 2
Linear Layer	4096 units
Linear Layer	4096 units
softmax	102 or 257 units

**Table 5:** Configuration of ssIncep-Net for the classification of Fashion MNIST and its variants.

Layers	Configuration for Each Layers
The First Convolutional Layer	size of kernels (64,3,3) with a stride of 1
The Second Convolutional Layer	size of kernels (128,3,3) with a stride of 1
Pooling Layer	size of pooling (2,2) with a stride of 1
The ssInception 3a block	size of kernels (64,1,1), (128,3,3) and (96,5,5) and size of pooling (3,3)
The ssInception 3b block	size of kernels (128,1,1), (192,3,3) and (96,5,5) and size of pooling (3,3)
Pooling Layer	size of pooling (3,3) with a stride of 2
The ssInception 4a block	size of kernels (192,1,1), (208,3,3) and (48,5,5) and size of pooling (3,3)
The ssInception 4b block	size of kernels (128,1,1), (192,3,3) and (96,5,5) and size of pooling (3,3)
The ssInception 4c block	size of kernels (246,1,1), (320,3,3) and (96,5,5) and size of pooling (3,3)
Pooling Layer	size of pooling (2,2) with a stride of 1
Linear Layer	4096 units
Linear Layer	4096 units
softmax	10 units

**Table 6:** Recognition error rates of the Fashion-MNIST variants.

Method	Dataset		
	Fashion-MNIST-Back-Rand (%)	Fashion-MNIST-Back (%)	Fashion-MNIST-Rot-Back (%)
DBN-3	13.77	17.38	55.27
GDBM-2	12.46	19.24	58.33
NIN	10.79	16.83	48.71
ResNet-10	9.64	13.19	46.02
Incep-Net	7.93	12.50	41.83
ssIncep-Net	6.15	9.97	35.74

## Data Availability

All data included in this study are available upon request by contact with the corresponding author.
